# Feed status and skin injury modulate immunopathology, global gene expression, and survival in channel catfish during virulent *Aeromonas hydrophila* infection

**DOI:** 10.3389/fimmu.2025.1642531

**Published:** 2025-07-22

**Authors:** Yesutor K. Soku, Miles D. Lange, Jason W. Abernathy, Nithin M. Sankappa, Craig A. Shoemaker, Karl Hayden, Linnea K. Andersen, Ida Phillips, Toufic Nashar, Temesgen Samuel, Abdelrahman Mohamed

**Affiliations:** ^1^ Department of Pathobiology, College of Veterinary Medicine, Tuskegee University, Tuskegee, AL, United States; ^2^ United States Department of Agriculture, Agricultural Research Service, Aquatic Animal Health Research Unit, Auburn, AL, United States; ^3^ Oak Ridge Institute for Science and Education (ORISE), Agricultural Research Services (ARS) Research Participation Program, Oak Ridge, TN, United States; ^4^ North Carolina Veterinary Diagnostic System, Raleigh, NC, United States

**Keywords:** inflammatory, pathogen recognition, gene expression, survival analysis, histopathology, innate immunity, gastrointestinal, aeromonas

## Abstract

**Introduction:**

Virulent *Aeromonas hydrophila* is a major pathogen in channel catfish (*Ictalurus punctatus*), that causes motile *Aeromonas* septicemia and significant economic losses. We investigated the effect of feeding status and skin integrity on the host immune response, disease survival, and gastrointestinal pathology following a vAh challenge.

**Methods:**

Using a bath immersion model, channel catfish were divided into four treatment groups: fin clipped and fed (FCF), fin clipped but not fed (FCN), not fin clipped but fed (NCF), and not fin clipped nor fed (NCN) alongside non-challenged control groups The FCF and NCF groups were fed 2 h prior to the challenge, but the FCN and NCN groups were not. Survival analysis, histopathological assessment, and RNA sequencing were conducted across groups at different time intervals throughout the vAh challenge.

**Results:**

Survival rates were lowest in the FCF and FCN groups (30% and 23% survival, respectively), suggesting that both feeding and skin damage contributed to disease severity. Histopathological analyses revealed more severe intestinal and gastric lesions in fed groups, characterized by epithelial necrosis, hemorrhage, and edema. Transcriptomic analysis among the groups identified significant differentially expressed genes associated with inflammation, apoptosis, and metabolic stress, with notable upregulation of interleukin 1-beta (*il-1β*), and complement C3 (*c3*). Gene ontology enrichment highlighted distinct immune activation patterns between fed and unfed groups, with enhanced pathogen recognition and pro-inflammatory responses in unfed fish.

**Discussion:**

These findings suggest feeding prior to infection may exacerbate disease pathology, potentially by creating a physiological state conducive to facilitate pathogen proliferation and dampened early immune responses, whereas short-term fasting appears to promote early immune activation. This study provides novel insights into the complex interplay between feed status, physical injury, and immune response to vAh infection.

## 1 Introduction

*Aeromonas hydrophila* is a Gram-negative, motile, rod-shaped bacterium widely found in aquatic environments that causes motile *Aeromonas* septicemia (MAS) in fish (1–3). Clinical signs can be acute, chronic, and latent, and infection manifests in various forms depending on the pathogen’s virulence factors, defense strategy, and stress level of the host (4). Over the last two decades, an *A. hydrophila* clonal strain emerged in the U.S., principally as isolated farm cases in 2004 (5). No other incidence was recorded until 2009, and since then, outbreaks from virulent *A. hydrophila* (vAh) strains typically affect market-size channel catfish (*Ictalurus punctatus*), with mortality ranging from 50-60% (6, 7). This pathogen accounts for $3.4 million annually in loss due to deaths and increased production costs, including the use of expensive antimicrobial feeds (8).

Previous work has investigated the portal of entry of vAh using bath immersion, intraperitoneal injection, and adipose fin (Af) clipping-immersion models (9–12). Quantification of vAh among internal organs demonstrates bacterial recovery in the gills, intestine, kidney, spleen, and adipose tissue 24 hours post-challenge (HPC) (13). Histological findings have been reported from natural and experimental vAh infections in channel catfish (7, 14), and histological changes were detected in the gills, intestine, stomach, kidney, and liver at 24 HPC (14), with lesions in the stomach and spleen significantly marked in natural infections (7). Af-clipping immersion models simulate a natural route of infection in channel catfish (13, 15); however, the characterization of pathological findings to assess the impact of nutritional status and fin clipping has not been reported.

Recent studies have also elucidated innate and adaptive immunity in channel catfish after intraperitoneal injection with vAh strain ML09-119 (16). Moreover, other studies have also investigated the catfish immune response to other bacterial, viral, and parasitic infections (17). These studies reveal that catfish combat bacterial infections utilizing their innate immune system, including pro-inflammatory cytokines and chemokines (16, 18). The transcriptomic analysis of the stomach and intestinal tissues of vAh-infected channel catfish following feeding or injury has not been evaluated.

Exploring the pathophysiology and immunobiology of motile *Aeromonas* septicemia caused by vAh in channel catfish following nutritional modulation and physical injury from fin clipping is critical to understanding how the fish responds to infection. This is particularly important in developing therapies, including vaccines and feed additives designed to confer protection against vAh. Based on the notable histological findings of degranulate eosinophilic granular cells and dendritic cells in the necrotic intestinal epithelium (14) and marked hemorrhage and edema in the submucosa and muscular of the stomach (7), we hypothesized that the gastrointestinal system plays a crucial role in the pathogenesis of vAh. We therefore aimed to investigate the pathology and global gene expression in vAh-infected channel catfish internal organs (intestine and stomach) following nutritional modulation and wounding.

## 2 Materials and methods

### 2.1 Ethics statement

All fish experiments were conducted at the USDA-ARS Aquatic Animal Health Research Unit (AAHRU) under an approved AAHRU Institutional Animal Care and Use Committee protocol and conformed to USDA-ARS Policies and Procedures 130.4.v5.

### 2.2 Fish source and housing conditions

Channel catfish weighing 35 ± 2 g were kept in a recirculating aquaculture system (RAS) at the AAHRU. These channel catfish were acclimated in dechlorinated water at 28 ± 2°C, pH at 7.5 ± 0.5, and dissolved oxygen at 7.0 ± 2.0 ppm for 14 days with daily monitoring of these water quality parameters. Fish in all treatment groups were fed the same pre-challenge commercial 32% protein 4.8 mm catfish pellets at 3% of their average body weight during acclimation and the day prior to the experiment. A photoperiod of 12:12 h light/darkness schedule was maintained. Aeration was supplied through air stones that were placed in each tank.

### 2.3 Bacteriology

An archived isolate of vAh ALG-15–097 was used for the study (9). The isolate was plated on tryptic soy agar supplemented with 5% sheep blood (Remel, Lenexa, KS) and incubated at 28 ± 2°C for 24 h. A single colony was then inoculated in tryptic soy broth (TSB) (Becton, Dickinson and Co., Sparks, MD) with 0.4 mM deferoxamine mesylate (DFO) (Sigma-Aldrich, St. Louis, MO) and incubated at 28 ± 2°C with 115 rpm constant shaking for 24 h. The overnight culture’s optical density (OD) at 540 nm was measured using an Ultrospec 2100 pro-UV/Visible Spectrophotometer (Pharmacia). Following this, triplicate plate count determined the concentration of ALG-15–097 colony-forming units (CFU) to be 1.5×10^9^ CFU/mL.

### 2.4 Experimental design

Channel catfish were transferred using nets into a 20 L container with RAS water mixed with 100 mg/L of Syncaine (MS-222) (Syndel, Ferndale, WA) buffered with sodium bicarbonate for sedation. To assess the effects of fed status, two groups of fish were fed 2 h before the challenge, whilst two groups were not. Similarly, to assess the effects of physical injury from tissue damage, the Af was clipped using scissors in two groups and left unclipped in two others. Briefly, upon full sedation, indicated by the absence of opercular movement after 5 minutes, the Af was clipped at its base using scissors, following the method outlined previously (12). No procedure other than anesthesia was conducted on the unclipped groups.

### 2.5 Treatment groups

1. FCF: These fish had their Af-clipped and fed 2 h before the challenge.
2. FCN: These fish had their Af-clipped and not fed 2 h before the challenge.
3. NCF: These fish had intact Af and fed 2 h before the challenge.
4. NCN: These fish had intact Af and not fed 2 h before the challenge.
5. Control1: These fish had their Af-clipped and exposed to sterile TSB.
6. Control2: These fish had intact Af and exposed to sterile TSB.

### 2.6 Bacterial challenge and survival curve analyses

Each treatment group included four replicate tanks. Following anesthesia, fish in each treatment group were split into four replicate tanks in a randomized, blinded manner (n = 25 fish per replicate tank). Each tank was 57 L equipped with air stones, filled with 10 L water and 100 mL of ALG-15–097 cell suspension (1.5×10^9^ CFU/mL) for a final concentration of 1.5×10^7^ CFU/mL (based on a pilot study that demonstrated this was an effective challenge dose). The controls were treated the same way, except they were exposed to 100 mL of sterile TSB (with 0.4 mM DFO). The fish remained in the 10 L bath for 1 h at which time the water flow was resumed at a rate of 0.5 L/min. The fish were monitored, and observations were recorded at 1 and 2 h intervals until 12 HPC. After that, observations were made twice daily. The experiment was concluded at 72 h. Dead fish were promptly removed from the tanks. About 20% of daily mortalities were sampled (liver) to confirm vAh as the cause of death. After the first day of the challenge, all fish groups were fed a commercial diet *ad libitum*. Survival data was analyzed using Kaplan-Meier log-rank survival analysis. Probabilities of *P* < 0.05 were considered statistically significant. All statistical tests were performed using GraphPad Prism version 10.3.1 (San Jose, CA).

### 2.7 Fish sampling for pathological analysis

Briefly, six randomly sampled fish from each treatment group were euthanized using an overdose of buffered Syncaine (>300 mg/L). A ventral midline incision was made from the vent to the pelvic girdle to expose the visceral organs. The dissected catfish were then placed in 10% neutral buffered formalin for 48 h to ensure proper tissue fixation, after which they were transferred to 70% ethanol for long-term preservation. This process was performed on fish sampled at 2, 4, and 8 HPC from each treatment group. A total of 72 fish were processed for analysis (n = 6/group/time). Additionally, 12 fish were randomly sampled from the two control treatment groups (n = 6/group) at 2 HPC to serve as controls for pathological investigation.

#### 2.7.1 Histopathology

After fixation, intestinal samples (fore- and hindgut) were collected and placed in cassettes for histological processing. This included tissue dehydration using alcohol, clearing in xylene, and embedding in paraffin wax. Thin sections, 5 µm thick, were then cut from the embedded tissues and stained with hematoxylin and eosin (H&E) and Gram stain. The stained sections were examined under a light microscope (Olympus BX41, Olympus America, Bartlett, TN) at total magnification of between 40-400x.

A semiquantitative grading system was developed to assess the pathological changes in the intestines of catfish sampled post-challenge. Severity scores were assigned based on the observed pathological changes, with mild, moderate or severe changes receiving 1, 2 and 3 points, respectively. Pathological features assessed included edema, hemorrhage, inflammation, and erosion/ulceration. The scores for changes within each individual tissue (stomach or intestine) were averaged, and a final score was assigned to each sample (**Table 1**). Statistical significance of the histopathology changes was assessed using nonparametric Kruskal-Wallis test and variables within scores assessed by Dunn’s *post-hoc* test (19).

**Table 1:** Semiquantitative grading system to assess the histological changes in the channel catfish intestine and stomach during the vAh challenge.

Treatments	Edema ^a^	Hemorrhage ^a^	Inflammation ^a^	Ulceration ^a^	Gastrointestinal Score Average ^a^
Control	0/0	0/0	0/0	0/0	0/0
FCF-2h	1.5/1.5	0/0	0.5/0.5	0/0	2/2
FCF-4h	2/2	0/0	2/2	0/0	4/4
FCF-8h	2.5/2.5	0/0	2/2	0/0	4.5/4.5
NCF-2h	1.5/1.5	0/0	0.5/0.5	0/0	2/2
NCF-4h	2/2	0/0	2/2	0/0	4/4
NCF-8h^*^	3/3	2/2	2/2	2/2	9/9
FCN-2h	1/1	0/0	0/0	0/0	1/1
FCN-4h	1/1	0/0	1/1	0/0	2/2
FCN-8h	1/1	0/0	1/1	0/0	2/2
NCN-2h	1/1	0/0	0/0	0/0	1/1
NCN-4h	1/1	0/0	0/0	0/0	1/1
NCN-8h	1/1	0/0	0/0	0/0	1/1

^a^The first value for each is the average in the stomach, and the second value is the average in the intestine.

^*^Only NCF-8h showed statistical significance with Dunn’s *post-hoc* test (p = 0.0088).

### 2.8 Fish sampling for RNA extraction and sequencing

The dissection and tissue sampling are the same as described above. After tissue sampling, each tissue (20–30 mg) was placed into 1 mL of RNA stabilization solution (RNAlater, ThermoFisher Scientific, Waltham, MA) and stored at -80°C freezer until needed. The same process was performed on challenged fish sampled at 2, 4, and 8 HPC from each treatment group. A total of 72 fish were processed for analysis (n = 6/group/time). Similarly, a total of 12 fish were randomly sampled from the two control treatment groups (n = 6/group) at 2 HPC for controls for RNA sequencing analysis.

### 2.9 RNA extraction and sequencing

Total RNA samples were assessed for quality and quantity using the 4200 TapeStation System with the RNA ScreenTape assay (Agilent Technologies, Santa Clara, CA) and a BioTek Cytation 1 Plate Reader with the BioTek Take3 Microvolume Plate (Agilent Technologies, Santa Clara, CA). Total RNA samples were then sent to a service provider (SeqMatic, Fremont, CA) for RNA sequencing on an Illumina NovaSeq X Instrument (San Diego, CA) in a 2 x 150 bp paired-end configuration with a target sequencing depth of > 25 M paired-end reads/sample. Sequencing libraries were made with the Illumina Stranded mRNA Ligation Prep Kit (Illumina, San Diego, CA) according to the manufacturer’s protocol.

### 2.10 Transcriptome analysis and bioinformatics

Raw, demultiplexed reads, with a minimum of 25 M paired-end reads/sample, were generated for analysis. Bioinformatics processing was carried out using OmicsBox software (20) and R-Bioconductor packages. The initial preprocessing of the raw FASTQ files involved quality control using FASTQC (21) and Trimmomatic (22) with default settings to remove low-quality bases, short reads, and Illumina adapter sequences. The channel catfish genome (Coco_2.0 assembly; GenBank accession GCA_001660625.3) was used for alignment. Quality-controlled (QC) reads were aligned using the STAR aligner (23) with 2-pass mapping and an overhang length of 149. The resulting BAM files were evaluated for alignment quality using RSeQC (24–26), generating metrics such as transcript integrity numbers (TINs). Gene-level counts were obtained from the QC-checked BAM files using HTSeq (27), with settings for strand-specific orientation, exon-based quantification, and union mode for overlapping reads. Differential expression analysis was conducted with edgeR (28), first, genes with low counts were filtered via the ‘filterByExpr’ function. Samples were then normalized using the Trimmed Mean of M values (TMM) method. The Quasi Likelihood F-test with the additional parameter of robust=true was used for pairwise comparisons, focusing on all four treatment groups and the control. Genes were classified as differentially expressed (DEGs) if they had an adjusted p-value below 0.05 and exhibited more than a 2-fold up- or downregulation compared to the control (*P*-adj < 0.05, FC > ± 2).

### 2.11 RT-qPCR validation

Select genes were independently assessed via reverse transcription quantitative polymerase chain reaction (RT-qPCR) to validate the RNA sequencing analyses. An aliquot of each total RNA sample used for RNAseq were also used for validation. cDNA synthesis was performed using the LunaScript^®^ RT SuperMix Kit (New England Biolabs, Ipswich, MA, United States). Reactions contained 4 μL of LunaScript RT SuperMix (5X) and 200 ng of template RNA, and the volume was adjusted to 20 μL with nuclease-free water. As a control, to rule out the presence of DNA in the samples, no-RT reactions were prepared for each of the samples, along with no-template controls (negative control). Reaction conditions for cDNA synthesis included primer annealing at 25°C for 2 min, cDNA synthesis at 55°C for 10 min and heat inactivation at 95°C for 1 min. RT-qPCR assays were performed using the Roche LightCycler 96 (Roche Diagnostics, Indianapolis, IN). Reactions were carried out in triplicate under the following conditions: 95°C for 15 s, followed by 45 cycles at 95°C for 15 s, 60°C for 30 s, followed by melting curve analysis. Cycle threshold (Ct) values were collected, and fold-change between each comparison was determined using the 2^-ΔΔCT^ method (29). *P*-values were calculated using a student’s t-test.

## 3 Results

### 3.1 vAh survival analysis

**Figure 1:**
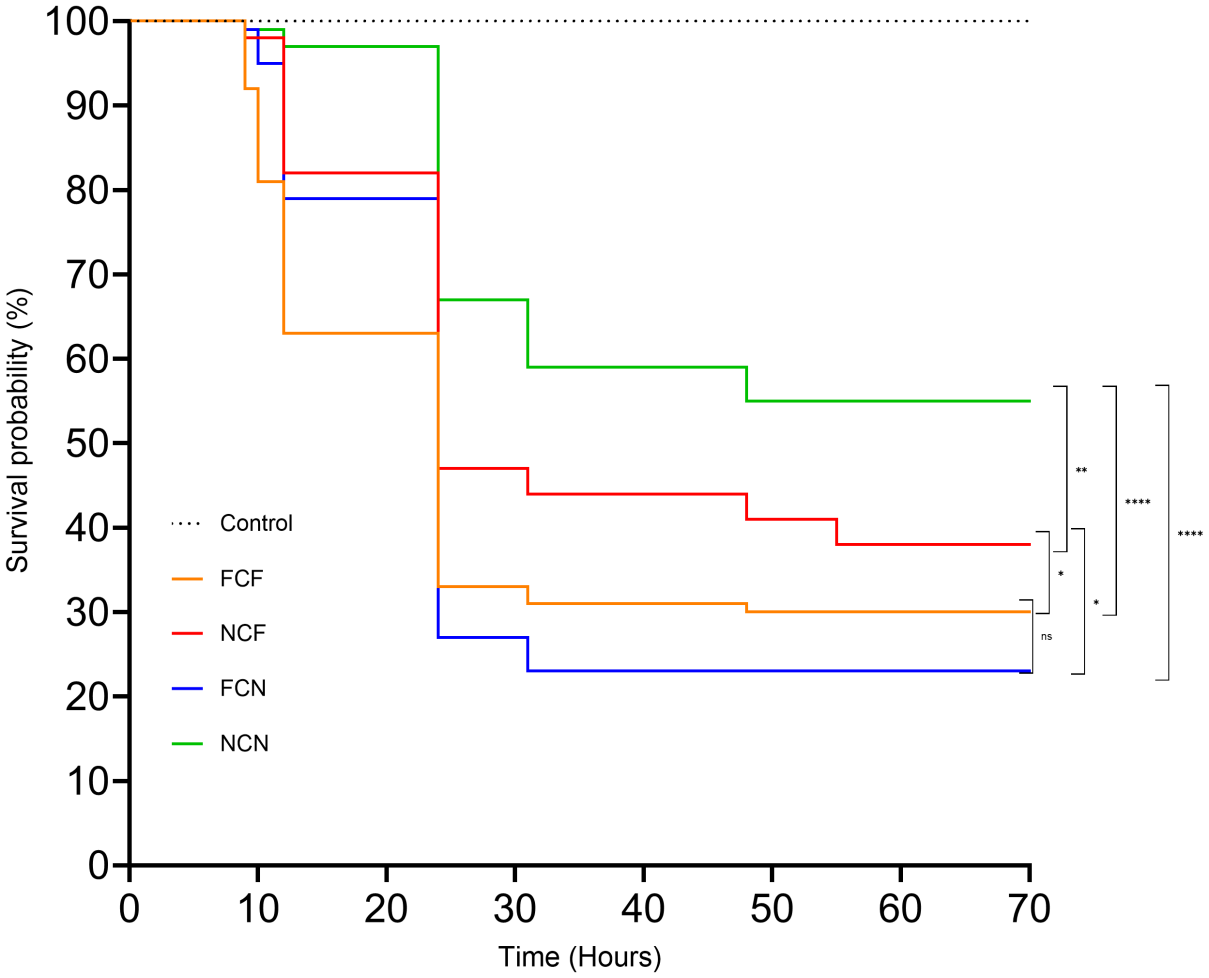
Kaplan-Meier survival curve of the treatment groups. FCF, Fin clip fed; NCF, No fin clip fed; FCN, Fin clip not fed; NCN, No fin clip not fed. The p-values for NCN vs. FCF, NCN vs. FCN, NCN vs. NCF, and, and NCF vs. FCN, and FCF vs. NCF were < 0.0001, < 0.0001, 0.0037,0.0244, and 0.0225 respectively. The p-value for FCF vs FCN was 0.6185. ns: Not significant (p > 0.05). *: Significant at p < 0.05. **: Significant at p < 0.01. ****: Significant at p < 0.0001.

In this study, we investigated the effects of fed status and physical injury on channel catfish during a vAh challenge. The fin clipped mock control incurred no mortality during the 72-h challenge. The FCN group experienced the highest mortality (77%), followed by FCF (70%), NCF (62%) and NCN (45%). Kaplan-Meier survival analysis showed a significantly higher survival rate in NCN compared to NCF (*P* < 0.01), FCF and FCN (*P* < 0.001) (**Figure 1**). NCF showed a significantly higher survival rate compared to FCF and FCN (*P* < 0.05). FCF and FCN survival rates were not significantly different from one another.

### 3.2 Gross and histopathological findings during vAh challenge

**Figure 2:**
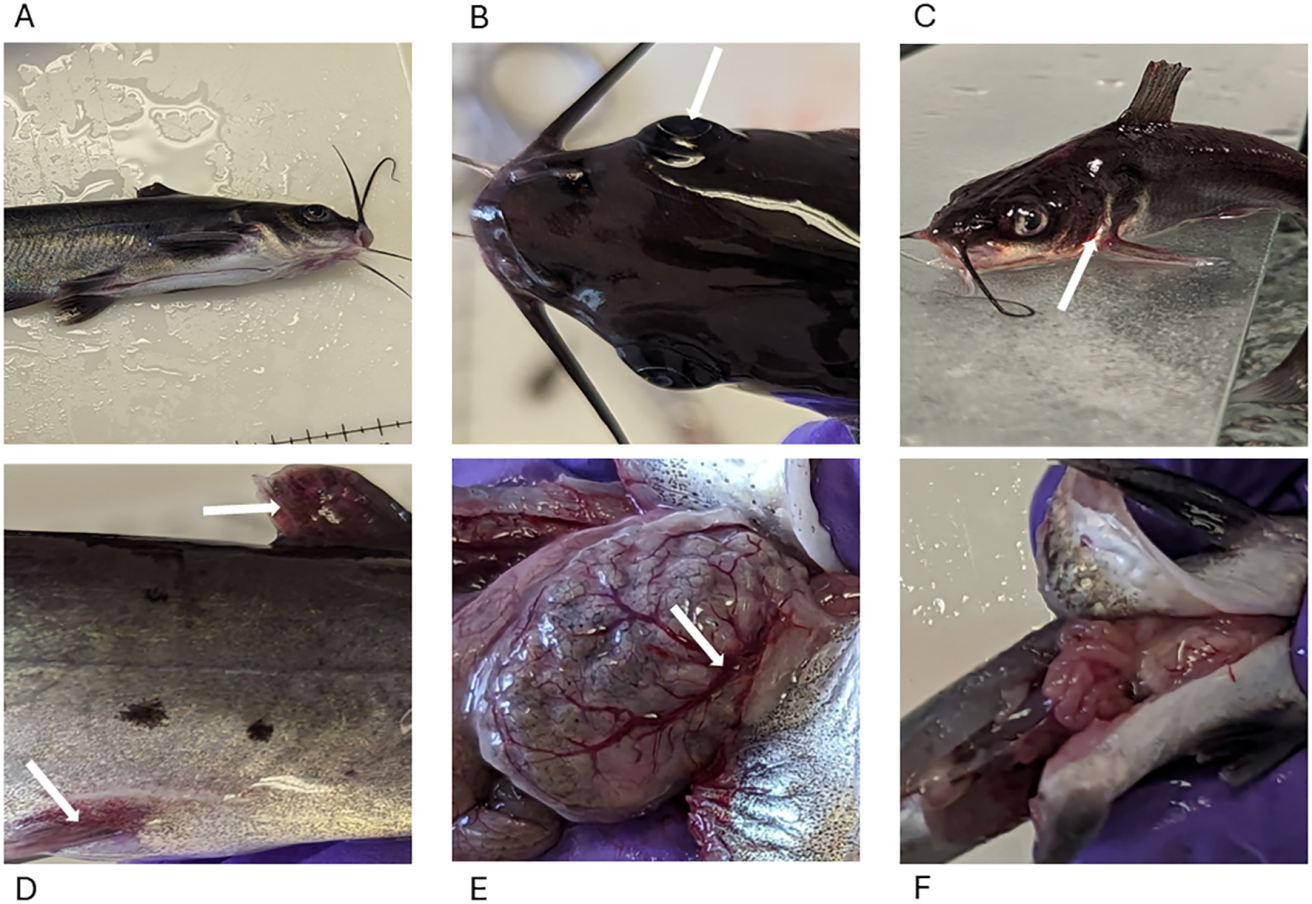
Lesions in MAS-infected channel catfish. **(A)** No marked external lesions were observed in fish from the control group. **(B)** Bilateral exophthalmia (arrow) with slight cellulitis on the skull region resembling the characteristic onset of skull lesions. **(C)** Reddening around the gills (arrow), mouth, and head region. **(D)** Erythema of the fins (dorsal and pelvic fins arrowed). **(E)** Dilated and engorged gastric blood vessels (arrow) 2 HPC in the fed treatment groups. **(F)** Gross appearance of unfed stomach 2 HPC in the unfed treatment group.

External lesions were observed on the surface of catfish by 2 HPC and included exophthalmia, iridial hemorrhage, reddened fins, and congestion, which are consistent with other findings (**Figures 2A–D**) (7, 30). Internally, the gastric blood vessels on the distended stomachs and intestines of the fed treatment groups were dilated, while the gastric vessels were not dilated in the unfed treatment groups (**Figures 2E, F**).

**Figure 3:**
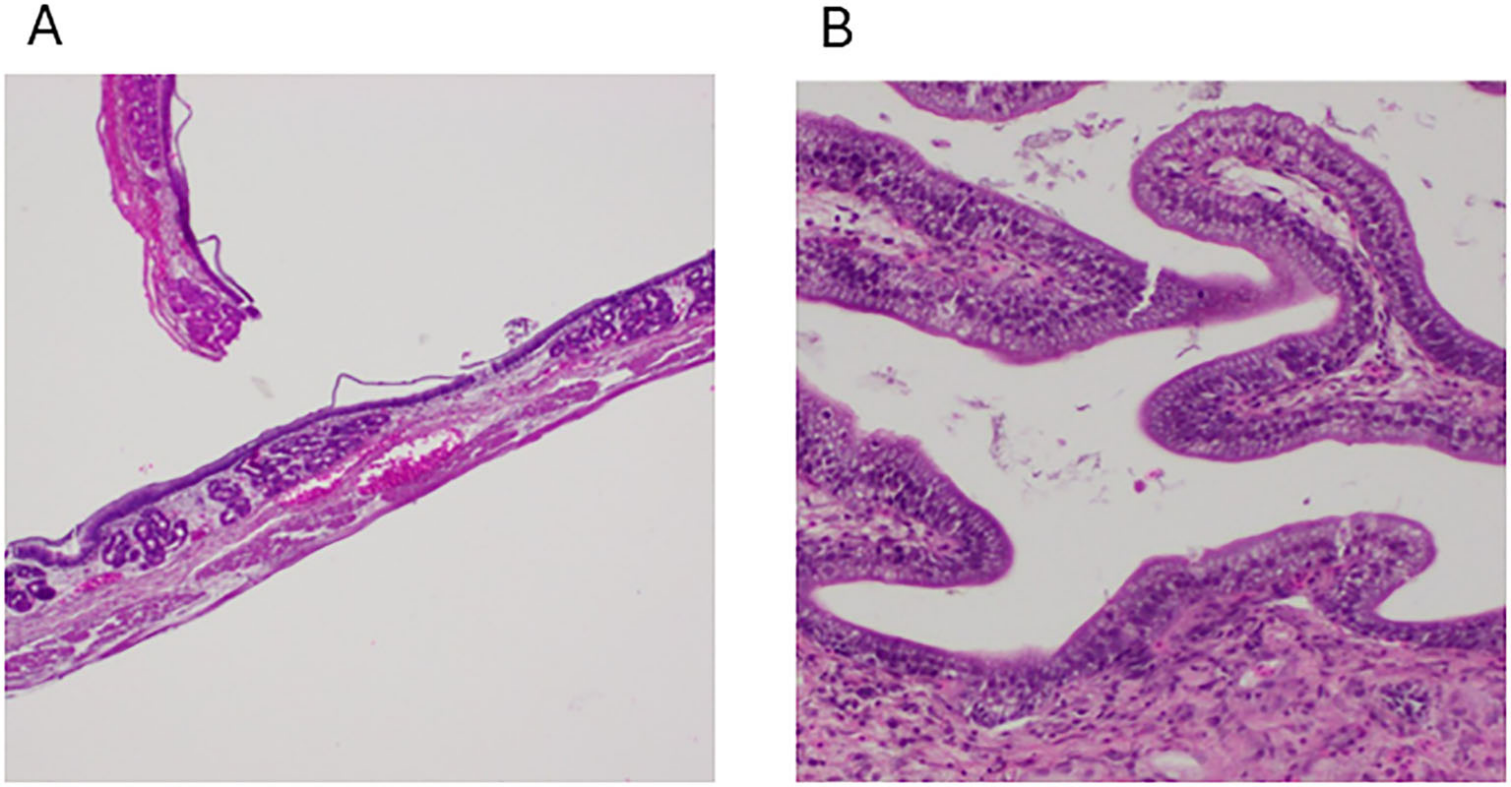
Photomicrographs from control group channel catfish. **(A)** Normal histology of the stomach showing all its structure, including the presence of gastric glands, 100x, H&E, FCF. **(B)** The intestines show normal structural arrangements, including the presence of goblet cells, 100x, H&E, NCF.

The control group presented a well-organized gastric structure in the stomach, maintaining the integrity of the mucosa and lamina propria (LP) with intact gastric glands and muscular and serosal layers (**Figure 3A**, **Table 1**). Similarly, the intestine displayed a preserved histoarchitecture with clearly delineated mucosal, submucosal, muscular, and serosal layers (**Figure 3B**).

**Figure 4:**
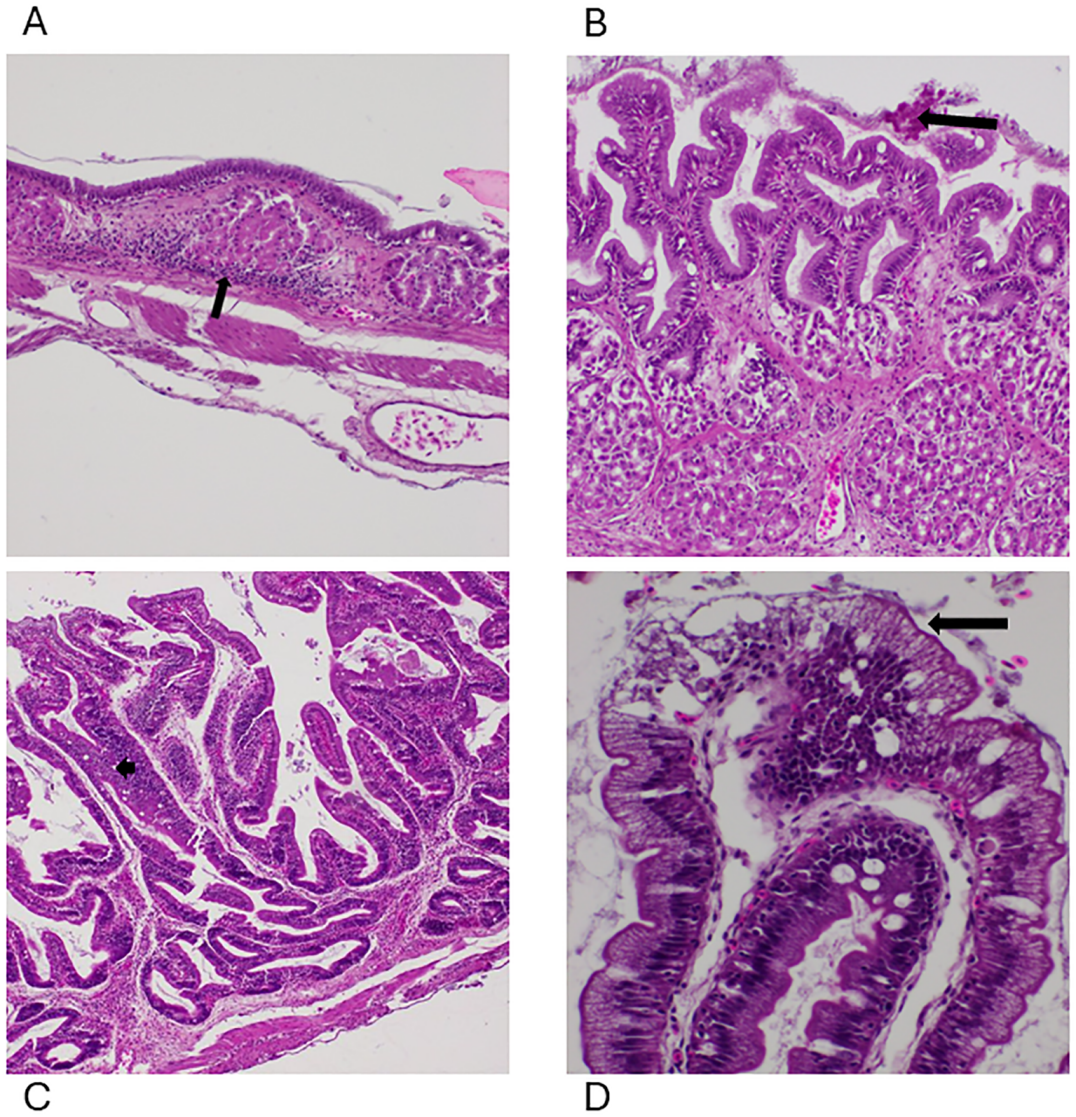
Photomicrographs of the lesions identified within the gastrointestinal system. **(A)** Moderate lymphoplasmacytic and granulocytic infiltration (arrow) in the stomach with mild and moderate edema in the FCF group at 4 HPC, 100x, H&E. **(B)** Multifocal erosions (arrow) to the superficial epithelium of the stomach with diffuse moderate to severe transmural edema in the NCF group at 8 HPC, 400x, H&E. **(C)** Moderate edema within the intestinal lamina propria (arrow) with a few lymphocytes and plasma cells (arrow) at 8 HPC, 200x, H&E, FCF. **(D)** Degenerative changes to the superficial epithelium of the intestine with diffuse severe transmural edema at 8 HPC, 200x, H&E, NCF.

Minimal to moderate edema was detected in the LP and submucosa of the stomach at 2 HPC (**Supplementary Figure S1**, **Table 1**). These lesions became more acute with the LP, and submucosa showing mild to moderate edema, with lymphoplasmacytic and granulocytic infiltration at 4 HPC (**Figure 4A**) and at 8 HPC, the stomach depicted multifocal hemorrhage and erosions to the superficial epithelium with diffuse moderate to severe transmural edema (**Figure 4B**). In the intestine, histopathological lesions were observed by 2 HPC and continued intervals through 8 HPC. Minimal to moderate edema within the LP was observed in the intestine at 2 HPC (**Supplementary Figure S2**, **Table 1**). At 4 HPC, the edema within the LP was mild to moderate, with the presence of lymphocytes and plasma cells (**Supplementary Figure S2**). At 8 HPC, degenerative changes to the superficial epithelium and hemorrhage were observed (**Figures 4C, D**).

Kruskal-Wallis test on the histology lesions revealed no statistically significant differences among the treatment groups. Additionally, *post-hoc* Dunn’s multiple comparisons test further confirmed that the majority of pairwise comparisons, including all comparisons between the control group and the treatment groups, were not significant, except for NCF-t8, (p = 0.0088).

### 3.3 Gastrointestinal gene expression analysis during vAh challenge

#### 3.3.1 Temporal changes and co-expression analysis of DEGs across treatments in the gastrointestinal tissues during vAh challenge

Within the fed group stomach, FCF expressed 3121 DEGs at 2 HPC, peaked with 3332 DEGs at 4 HPC, and then decreased to 3207 DEGs at 8 HPC, whereas NCF expressed 200 DEGs at 2 HPC, rapidly increased to 1252 at 4 HPC and reduced to 1195 at 8 HPC. In the unfed groups, FCN peaked at 2 HPC with 4413 DEGs, decreasing to 3938 DEGs at 4 HPC, and 3709 at 8 HPC, while NCN showed 2618 DEGs at 2 HPC, peaked with 2792 DEGs at 4 HPC and reduced to 2443 DEGs at 8 HPC (**Supplementary Table S1**).

**Figure 5:**
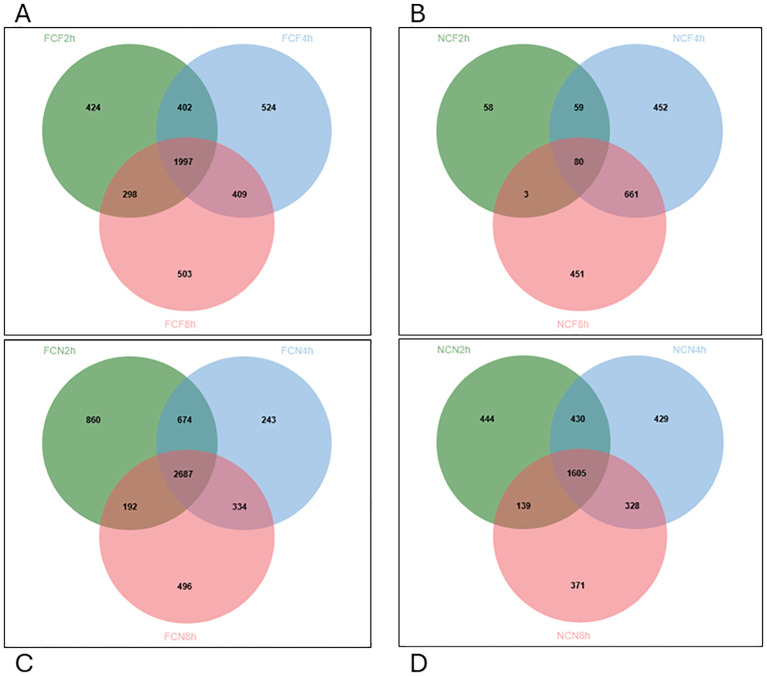
Venn diagram displaying unique and commonly expressed DEGs at 2, 4, 8 HPC in the different treatment groups of the stomach (*P* < 0.05). **(A)** Fin clip fed (FCF). **(B)** No fin clip fed (NCF). **(C)** Fin clip not fed (FCN). **(D)** No fin clip not fed (NCN).

Venn diagram analysis revealed that FCF had 1997 DEGs co-expressed across all three-time intervals, 402 co-expressions between 2 and 4 HPC, 409 between 4 and 8 HPC, and in the NCF group, 80 co-expressions at all three-time intervals, 59 between 2 and 4 HPC and 441 between 4 and 8 HPC (**Figures 5A, B**). Likewise, in FCN, 2687 DEGs were co-shared at all three intervals, 674 between 2 and 4 HPC and 334 between 4 and 8 HPC. In the NCN group, 1605 DEGs were co-shared at all three-time intervals, 430 between 2 and 4 HPC and 328 between 4 and 8 HPC intervals (**Figures 5C, D**, **Supplementary Table S1**).

Within the fed group intestine, DEGs peaked at 4 HPC. At 2 HPC, 410 DEGs were identified in the FCF group, while 916 DEGs were found in the NCF group. A similar trend was observed at 4 and 8 HPC, with fewer FCF DEGs than NCF. In contrast, the unfed groups exhibited more DEGs, with 1803 DEGs in the FCN group at 2 HPC, increasing to 2013 DEGs at 4 HPC and 2007 DEGs at 8 HPC. A similar trend of peak expression at 4 HPC was observed in the NCN group (**Supplementary Table S2**).

**Figure 6:**
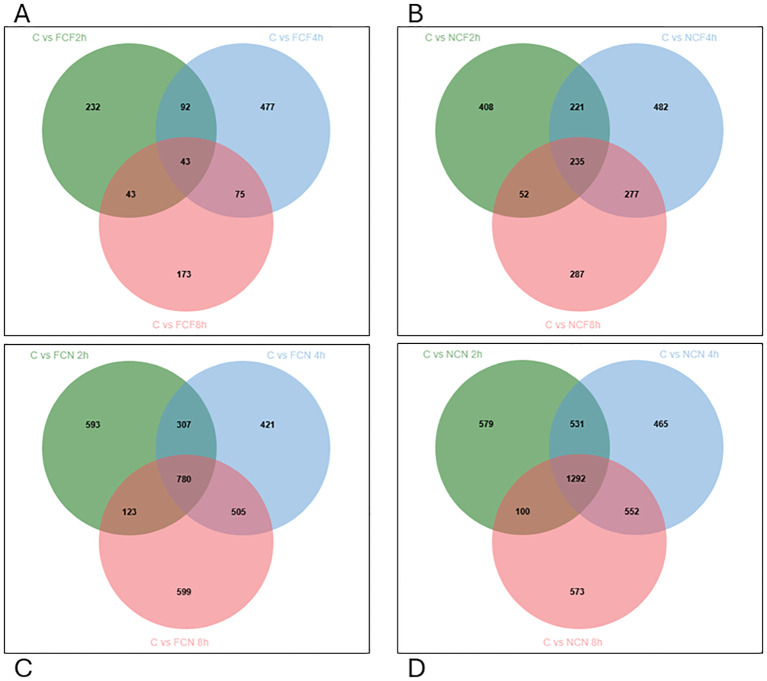
Venn diagram displaying unique and commonly expressed DEGs at 2, 4 and 8 HPC in the different treatment groups of the intestine (*P* < 0.05). **(A)** Fin clip fed (FCF). **(B)** No fin clip fed (NCF). **(C)** Fin clip not fed (FCN). **(D)** No fin clip not fed (NCN).

Venn diagram analysis identified overlapping DEGs across time intervals. In the FCF group, 92 DEGs were co-expressed between 2 and 4 HPC, 75 DEGs between 4 and 8 HPC, and 43 DEGs were common across all time intervals (**Figure 6A**). In the NCF group, 221 DEGs were shared between 2 and 4 HPC, 277 between 4 and 8 HPC, and 235 DEGs persisted across all time intervals (**Figure 6B**). Similarly, in the unfed groups, FCN exhibited 307 co-expressed DEGs between 2 and 4 HPC, 505 between 4 and 8 HPC, and 780 DEGs across all time intervals, and in NCN, 531 DEGs were shared between 2 and 4 HPC, 552 between 4 and 8 HPC, and 1292 were consistently expressed across time intervals (**Figures 6C, D**, **Supplementary Table S2**).

#### 3.3.2 Gene ontology of gastrointestinal tissues during vAh challenge

In the FCF, the top GO terms that were over-represented in the stomach included processes associated with mitochondrion function (GO:0005739), oxidoreductase activity (GO:0016491) and organic acid metabolism (GO:004331) (**Table 2**, **Supplementary Table S3**). In the intestine, peptidyl-proline modification (GO:0018208), ferrous iron binding (GO:0008198) and oxidoreductase activity (GO:0016491) were over-represented.

**Table 2:** The top ten gene ontology enrichment groups identified among DEGs shared at the (2-, 4- and 8-h) time intervals of fin-clipped fed and not-fed catfish gastrointestinal tissues during the vAh challenge.

Trt.	Tissue	GO Group^a^	GO ID	GO Term	Designation^b^	*P*-value
FCF	Stomach	CC	GO:0005739	mitochondrion	OVER	8.4E-66
		BP	GO:0044281	small molecule metabolic process	OVER	3.3E-45
		CC	GO:0005759	mitochondrial matrix	OVER	1.2E-32
		BP	GO:0008152	metabolic process	OVER	6.2E-32
		MF	GO:0016491	oxidoreductase activity	OVER	3.5E-31
		CC	GO:0005737	cytoplasm	OVER	9.9E-30
		BP	GO:0019752	carboxylic acid metabolic process	OVER	2.6E-29
		BP	GO:0043436	oxoacid metabolic process	OVER	1.5E-28
		CC	GO:0043231	intracellular membrane-bounded organelle	OVER	1.7E-28
		BP	GO:0006082	organic acid metabolic process	OVER	1.9E-28
	Intestine	BP	GO:0018126	protein hydroxylation	OVER	0.00216
		BP	GO:0018401	peptidyl-proline hydroxylation to 4-hydroxy-L-proline	OVER	0.00411
		MF	GO:0031545	peptidyl-proline 4-dioxygenase activity	OVER	0.00411
		BP	GO:0018208	peptidyl-proline modification	OVER	0.00411
		MF	GO:0019842	vitamin binding	OVER	0.00505
		MF	GO:0031543	peptidyl-proline dioxygenase activity	OVER	0.00505
		BP	GO:0019471	4-hydroxyproline metabolic process	OVER	0.00505
		BP	GO:0019511	peptidyl-proline hydroxylation	OVER	0.00533
		MF	GO:0008198	ferrous iron binding	OVER	0.00614
		MF	GO:0016491	oxidoreductase activity	OVER	0.00749
FCN	Stomach	CC	GO:0043231	intracellular membrane-bounded organelle	OVER	2.0E-73
		CC	GO:0005739	mitochondrion	OVER	2.8E-69
		CC	GO:0043227	membrane-bounded organelle	OVER	2.8E-65
		CC	GO:0005622	intracellular anatomical structure	OVER	3.5E-52
		CC	GO:0043229	intracellular organelle	OVER	8.6E-52
		BP	GO:0008152	metabolic process	OVER	4.2E-48
		CC	GO:0043226	organelle	OVER	4.7E-44
		CC	GO:0005737	cytoplasm	OVER	4.3E-42
		CC	GO:0005759	mitochondrial matrix	OVER	9.0E-39
		CC	GO:0098798	mitochondrial protein-containing complex	OVER	5.1E-38
	Intestine	CC	GO:0043231	intracellular membrane-bounded organelle	OVER	9.0E-10
		CC	GO:0005783	endoplasmic reticulum	OVER	4.4E-09
		CC	GO:0043227	membrane-bounded organelle	OVER	3.9E-08
		CC	GO:0042995	cell projection	OVER	5.0E-08
		CC	GO:0005737	cytoplasm	OVER	6.8E-08
		CC	GO:0120025	plasma membrane bounded cell projection	OVER	1.1E-07
		CC	GO:0005739	mitochondrion	OVER	3.9E-07
		MF	GO:0016491	oxidoreductase activity	OVER	3.9E-07
		BP	GO:0044281	small molecule metabolic process	OVER	5.1E-07
		CC	GO:0005622	intracellular anatomical structure	OVER	5.8E-07

^a^BP, Biological Process; MF, Molecular Function; CC, Cellular Component.

^b^OVER indicates a significant (adj. *P*-value < 0.05; Fisher’s Exact Test) enrichment of GO terms in each group when compared to the annotated channel catfish genome.

In the FCN, the top GO terms that were over-represented in the stomach included processes associated with mitochondrion function (GO:0005739), metabolic process (GO:0008152) and mitochondrial matrix (GO:0005759) (**Table 2**, **Supplementary Table S3**). In the intestine, mitochondrion function (GO:0005739), oxidoreductase activity (GO:0016491) and small molecule metabolic process (GO:0044281) were over-represented.

In NCF, the stomach over-represented processes associated with oxidoreductase activity (GO:0016491), catalytic activity (GO: 0003824) and detoxification (GO:0098754) were identified (**Table 3**, **Supplementary Table S3**). In the intestine, cell cycle (GO:0007049), regulation of cell cycle (GO:0051726) and response to stress (GO:0006950) were over-represented. In the NCN, the top GO terms that were over-represented in the stomach included processes associated with metabolic process (GO:008152), oxidoreductase activity (GO:0016491) and aerobic respiration (GO:0009060) (**Table 3**, **Supplementary Table S3**). In the intestine, mitochondrion function (GO:0005739), aerobic respiration (GO:0009060) and oxidoreductase activity (GO:0016491) were over-represented.

**Table 3:** The top ten gene ontology enrichment groups identified among DEGs shared at the (2-, 4- and 8-h) time intervals of non-clipped fed and not-fed catfish gastrointestinal tissues during the vAh challenge.

Trt.	Tissue	GO Group^a^	GO ID	GO Term	Designation^b^	*P*-value
NCF	Stomach	MF	GO:0016491	oxidoreductase activity	OVER	3.60E-08
		MF	GO:0016209	antioxidant activity	OVER	0.00224
		MF	GO:0003824	catalytic activity	OVER	0.00224
		BP	GO:0098869	cellular oxidant detoxification	OVER	0.00224
		BP	GO:1990748	cellular detoxification	OVER	0.00224
		BP	GO:0071450	cellular response to oxygen radical	OVER	0.00224
		BP	GO:0071451	cellular response to superoxide	OVER	0.00224
		BP	GO:0019430	removal of superoxide radicals	OVER	0.00224
		BP	GO:0098754	detoxification	OVER	0.00439
		BP	GO:0055086	nucleobase-containing small molecule metabolic process	OVER	0.00501
	Intestine	BP	GO:0007049	cell cycle	OVER	1.70E-06
		BP	GO:0000278	mitotic cell cycle	OVER	8.20E-05
		BP	GO:0051726	regulation of cell cycle	OVER	8.40E-05
		BP	GO:0045786	negative regulation of cell cycle	OVER	6.00E-04
		BP	GO:0010948	negative regulation of cell cycle process	OVER	6.90E-04
		BP	GO:0007093	mitotic cell cycle checkpoint signaling	OVER	7.30E-04
		CC	GO:0005694	chromosome	OVER	0.00128
		BP	GO:0006950	response to stress	OVER	0.00568
		BP	GO:0000075	cell cycle checkpoint signaling	OVER	0.00568
		MF	GO:1901363	heterocyclic compound binding	OVER	0.00568
NCN	Stomach	CC	GO:0005739	mitochondrion	OVER	9.90E-53
		CC	GO:0043231	intracellular membrane-bounded organelle	OVER	8.20E-36
		BP	GO:0008152	metabolic process	OVER	6.00E-33
		BP	GO:0022900	electron transport chain	OVER	1.90E-32
		CC	GO:0043227	membrane-bounded organelle	OVER	3.70E-32
		BP	GO:0045333	cellular respiration	OVER	2.10E-31
		MF	GO:0016491	oxidoreductase activity	OVER	3.60E-31
		CC	GO:0005737	cytoplasm	OVER	6.50E-30
		BP	GO:0009060	aerobic respiration	OVER	7.20E-30
		BP	GO:0006119	oxidative phosphorylation	OVER	2.70E-29
	Intestine	CC	GO:0005739	mitochondrion	OVER	7.50E-42
		CC	GO:0043231	intracellular membrane-bounded organelle	OVER	5.00E-31
		BP	GO:0045333	cellular respiration	OVER	5.80E-30
		BP	GO:0009060	aerobic respiration	OVER	1.50E-27
		CC	GO:0005737	cytoplasm	OVER	1.90E-27
		CC	GO:0043227	membrane-bounded organelle	OVER	1.20E-26
		BP	GO:0044281	small molecule metabolic process	OVER	2.40E-25
		MF	GO:0016491	oxidoreductase activity	OVER	4.40E-24
		BP	GO:0022900	electron transport chain	OVER	6.20E-24
		BP	GO:0006091	generation of precursor metabolites and energy	OVER	7.40E-24

^a^BP, Biological Process; MF, Molecular Function; CC, Cellular Component.

^b^OVER indicates a significant (adj. *P*-value < 0.05; Fisher’s Exact Test) enrichment of GO terms in each group when compared to the annotated channel catfish genome.

To further assess potential associations between DEGs among the different time intervals and their relationship to disease progression, we selected enrichment terms dealing with immune function (**Table 4**, **Supplementary Table S3**). Several GO terms identified for antigen processing and presentation (GO:0019884, GO:0042590, GO:0002479 and GO:0002474) were present in the stomach of FCF, FCN and NCN were over-represented. Apoptotic pathways (GO:0006915, GO:2000107 and GO:2000425 were identified in the stomach of FCF and the stomach and intestine of FCN. Different cell signaling and receptor activity terms such as cell surface receptor signaling, cytokine production involved in immune response, receptor tyrosine kinase binding, signaling and transmembrane signaling activity (GO:0007166, GO:0002367, GO:0030971, GO:0038023 and GO:0004888) were identified in the stomach of FCF, FCN and NCN. Inflammatory keywords and pathways (GO:0006954, GO:0032615 and GO:0032655) were identified in the stomach of FCF and FCN. Neutrophil activation, chemotaxis, and killing activity (GO:042119, GO:0030593, GO:0070945 were found in the stomach of FCF, FCN. The presence of reactive oxygen species processes (GO:0072593, GO:2000377 and GO:0000302) was prevalent in the stomach of FCF and FCN and in the NCN stomach and intestine.

**Table 4:** Selected gene ontology terms related to immune function identified among treatment DEGs shared at the (2-, 4- and 8-h) time intervals in the catfish gastrointestinal tissues during the vAh challenge.

GO Term	Trt. (Tissue)^a^	*P*-value^b^	Designation
Antigen processing and presentation of exogenous antigen (GO:0019884)	FCF (St)	0.03591	OVER
	FCN (St)	0.02229	OVER
Antigen processing and presentation of exogenous peptide antigen via MHC class I (GO: 0042590)	FCF (St)	9.60E-04	OVER
	FCN (St)	0.0037	OVER
	NCN (St)	0.03434	OVER
Antigen processing and presentation of exogenous peptide antigen via MHC class I, TAP-dependent (GO:0002479)	FCF (St)	0.01953	OVER
	FCN (St)	0.00562	OVER
	NCN (St)	0.01048	OVER
Antigen processing and presentation of peptide antigen via MHC class I (GO:0002474)	FCF (St)	0.0044	OVER
	FCN (St)	0.00373	OVER
	NCN (St)	0.0093	OVER
Apoptotic process (GO:0006915)	FCN (St)	0.02122	OVER
Cell surface receptor signaling pathway (GO:0007166)	NCN (St)	0.00427	UNDER
Cellular response to reactive oxygen species (GO:0034614)	FCF (St)	0.00636	OVER
	FCN (St)	0.01601	OVER
	NCN (Int)	0.03556	OVER
Complement component C1q complex binding (GO:0001849)	NCN (St)	0.00427	OVER
Cytokine production involved in immune response (GO:0002367)	FCF (St)	0.03404	OVER
Immune response-regulating cell surface receptor signaling pathway (GO:0002768)	NCN (St)	0.01853	UNDER
Inflammatory response (GO:0006954)	FCF (St)	0.03159	OVER
Interleukin-12 production (GO:0032615)	FCN (St)	0.04908	OVER
Negative regulation of leukocyte apoptotic process (GO:2000107)	FCN (Int)	0.04053	OVER
Neutrophil activation (GO:0042119)	FCN (St)	0.04951	OVER
Neutrophil chemotaxis (GO:0030593)	FCN (St)	0.04474	OVER
Neutrophil-mediated killing of bacterium (GO:0070944)	FCF (St)	0.03478	OVER
Neutrophil-mediated killing of gram-negative bacterium (GO:0070945)	FCF (St)	0.03478	OVER
Positive regulation of MyD88-dependent toll-like receptor signaling pathway (GO: 0034126)	FCN (St)	0.02912	OVER
Reactive oxygen species metabolic process (GO:0072593)	NCN (St)	4.30E-11	OVER
	FCN (St)	8.10E-08	OVER
	FCF (St)	2.40E-06	OVER
	NCN (Int)	0.00329	OVER
Receptor tyrosine kinase binding (GO: 0030971)	FCN (St)	0.02912	OVER
Regulation of apoptotic cell clearance (GO:2000425)	FCN (St)	0.04298	UNDER
Regulation of interleukin-12 production (GO:0032655)	FCN (St)	0.03195	OVER
Regulation of reactive oxygen species metabolic process (GO:2000377)	FCF (St)	4.49E-04	OVER
	FCN (St)	0.01771	OVER
	NCN (St)	0.00252	OVER
Response to reactive oxygen species (GO:0000302)	NCN (Int)	0.00745	OVER
	FCF (St)	0.00669	OVER
	FCN (St)	0.02473	OVER
Signaling receptor activity (GO:0038023)	NCN (St)	0.01683	UNDER
Toll-like receptor 3 signaling pathway (GO:0034138)	FCF (St)	0.03812	OVER
Transmembrane signaling receptor activity (GO:0004888)	NCN (St)	9.60E-04	UNDER

^a^Tissue description are stomach (St) and intestine (Int). ^b^OVER or UNDER representation of GO terms when compared to the annotated channel catfish genome (adj. *P*-value < 0.05; Fisher’s Exact Test).

#### 3.3.3 Immune surveillance in the gastrointestinal tissues during vAh challenge

**Figure 7:**
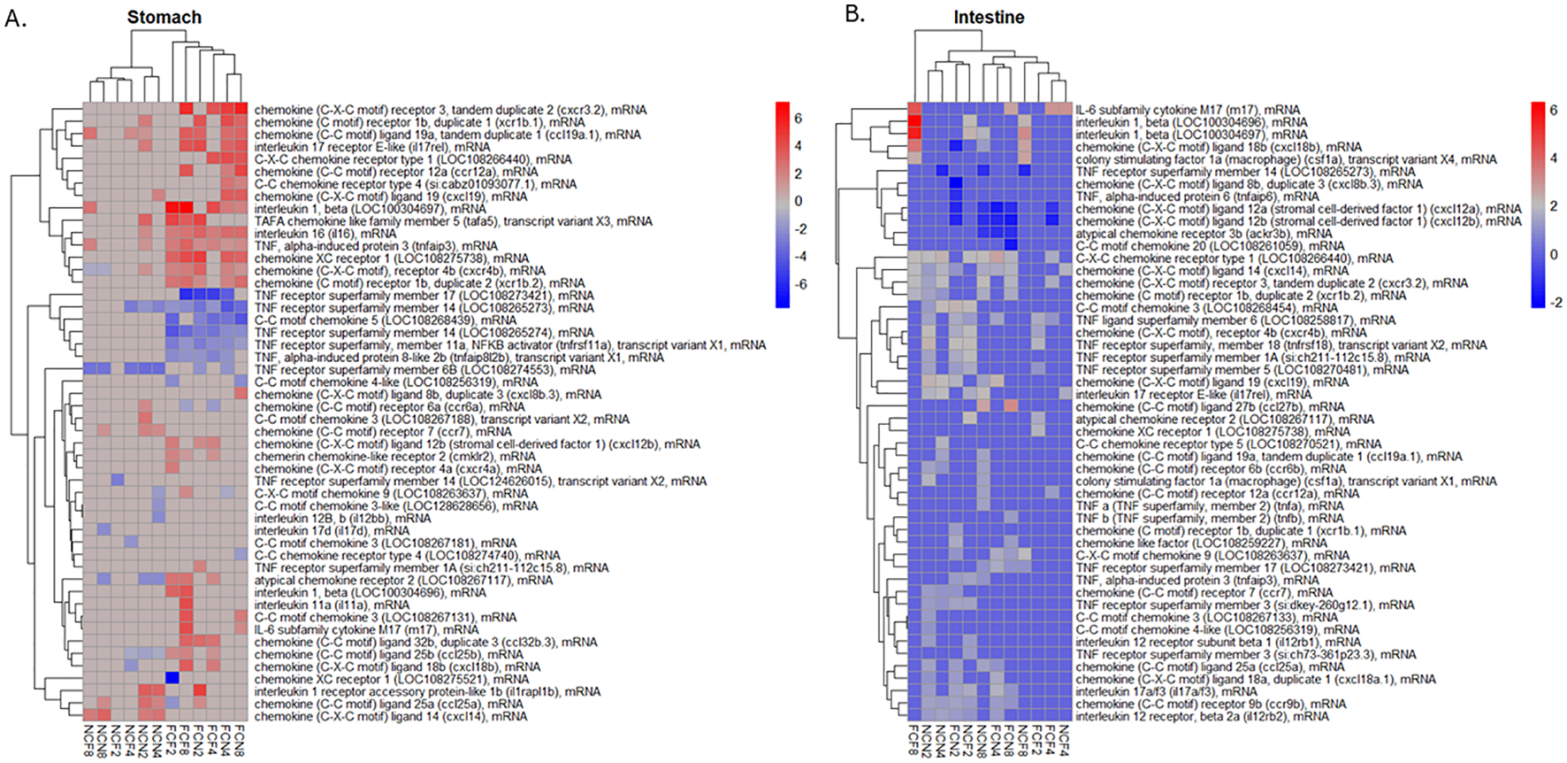
Heatmap of the top 50 DEGs; up- or down-regulated related to a pro-inflammatory response were hierarchically clustered by logFC (*P <*0.05) for the **(A)** stomach and **(B)** intestine at 2, 4 and 8 HPC. Fin clip-fed (FCF), no fin clip fed (NCF), fin clip not fed (FCN), and no fin clip not fed (NCN). LogFC values are shown as a color scale at the right of each heat map.

Many pro-inflammatory cytokines, interleukins, chemokines, and TNF receptors were identified in the stomach and intestine (**Supplementary Table S4**). Visualization of the top 50 up- or -down-regulated DEGs by the absolute value of logFC is shown in **Figure 7**. The upregulated gene expression of the FCF stomach included chemokine ligand 18B (*cxcl18b*) at 4 and 8 HPC, interleukin 1-beta (*il-1β*) and TNF alpha-induced protein 6 (*tnfaip6*) at 2, 4 and 8 HPC along with several other chemokine genes (**Figure 7A**, **Supplementary Table S4**). The downregulated gene expression included TNF receptor superfamily member 11a NFKB activator (*tnfrsf11a*) and TNF receptor superfamily member 14 (*tnfrsf14 LOC108265273*) at 2, 4 and 8 HPC along with other TNF receptors. The upregulated gene expression of the FCN stomach identified *il-1β LOC100304697* at 4- and 8-HPC and chemokine receptor 4b (*cxcr4b*) at 2, 4 and 8 HPC (**Figure 7A**, **Supplementary Table S4**). The downregulated gene expression included both the TNF receptor superfamily member 11a NFKB activator (*tnfrsf11a*) and *tnfrsf14 LOC108265273* at 2, 4 and 8 HPC.

The expression of the pro-inflammatory cytokines was much more limited in the NCF and NCN. The upregulated genes in the NCF stomach were *il-1β LOC100304697* and chemokine ligand 14 (*cxcl14*) at 8 HPC. The downregulated genes were *tnfrsf14 LOC124626015* and TNF receptor superfamily member 6B (*tnfrsf6b LOC108274553*) at 2 and 8 HPC, respectively (**Figure 7A**, **Supplementary Table S4**).

A list of the DEGs in the intestine was also examined in a similar manner (**Figure 7B**, **Supplementary Table S4**). The upregulated gene expression in the FCF intestine included two different interleukin 1-beta genes (*LOC100304696* and *LOC100304697*), followed by the chemokine ligand 18B (*cxcl18b*) all at 8 HPC. The most downregulated genes were chemokine ligand 12b (*cxcl12b*) and chemokine ligand 12a (*cxcl12a*) at 2 and 8 HPC, respectively. The FCN intestine limited upregulated genes included chemokine 3 (*LOC108268454*) at 2 HPC and IL-6 subfamily cytokine M17 (*m17*) at 8 HPC. The downregulated genes were chemokine ligand 12a (*cxcl12a*) and chemokine ligand 12b (*cxcl12b*) each at 2, 4 and 8 HPC.

The upregulated gene expression in the NCF intestine included two different *il-1β* genes (*LOC100304696* and *LOC100304697)* identified at 2 and 8 HPC, followed by the *cxcl18b* at 8 HPC. The downregulated gene identified was *tnfrsf14 LOC108265273* at 8 HPC. The upregulated gene expression in the NCN intestine included the chemokine ligand 14 (*cxcl14*) and chemokine receptor type 1 (*LOC108266440*), each at 2, 4 and 8 HPC. The downregulated gene identified was chemokine ligand 12a (*cxcl12a*) and chemokine ligand 12b (*cxcl12b*) at 8 HPC.

#### 3.3.4 Complement and apoptotic activity in the gastrointestinal tissues during vAh challenge

**Figure 8:**
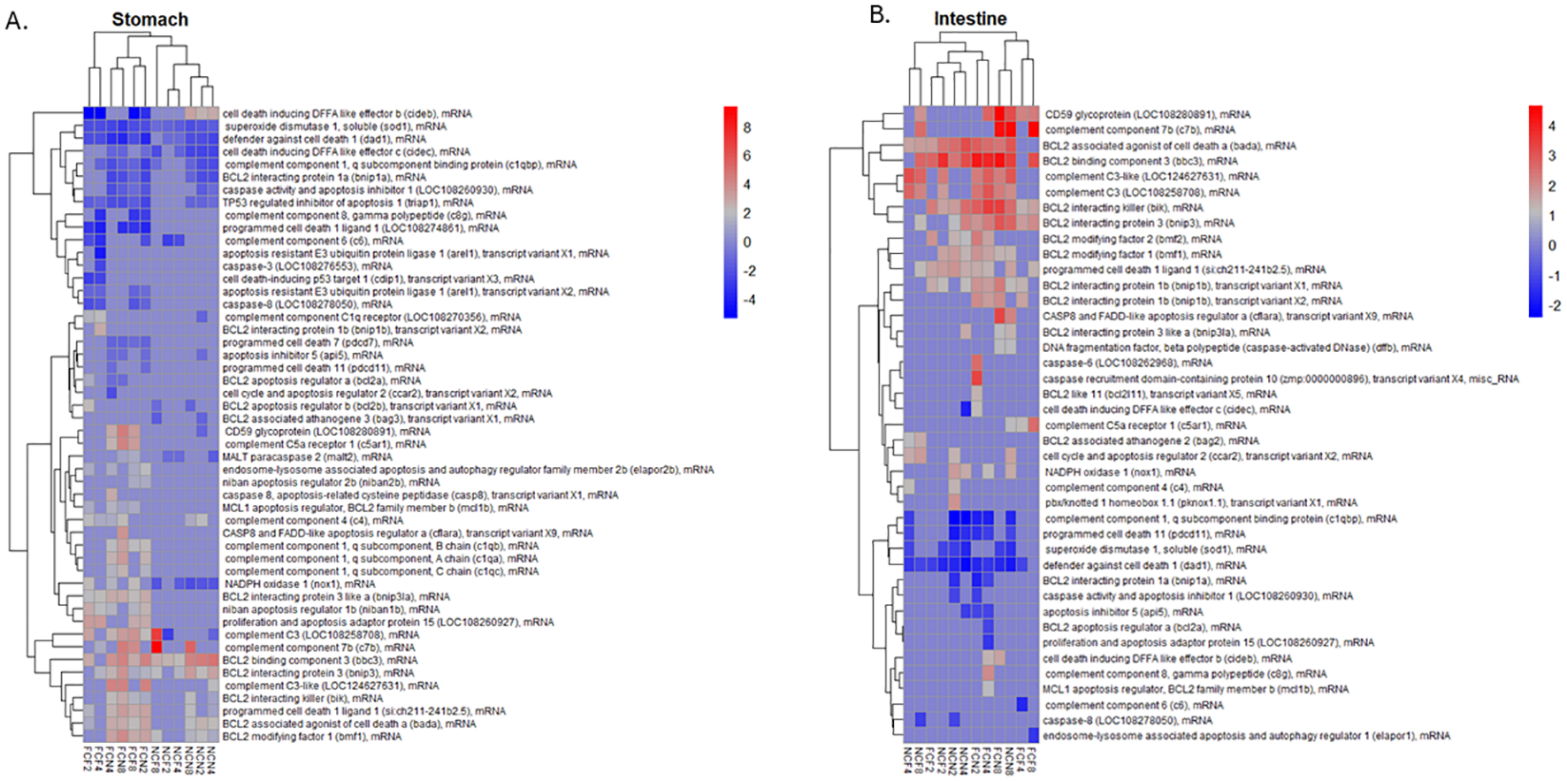
Heatmap of the top 50 DEGs; up- or down-regulated related to complement and apoptosis were hierarchically clustered by logFC (*P <*0.05) for the **(A)** stomach and **(B)** intestine at 2, 4 and 8 HPC. Fin clip fed (FCF), no fin clip fed (NCF), fin clip not fed (FCN), and no fin clip not fed (NCN). LogFC values are shown as a color scale at the right of each heat map.

To further examine the status of cell homeostasis in the different tissues, we evaluated the expression of individual DEGs associated with cell death among the different time intervals post-vAh challenge (**Figure 8A**, **Supplementary Table S4**). The upregulated gene expression of the FCF stomach included complement component 7b (*c7b*), complement C3 (*c3 LOC108258708*), and complement C5a receptor 1 (*c5ar1*) at 8 HPC. The downregulated gene expression in FCF stomach included apoptosis-resistant E3 ubiquitin protein ligase 1 (*arel1*) and programmed cell death 1 ligand 1 (*pd-1l LOC108274861*) at 4 HPC, and cell death inducing DFFA like effector b (*cideb*) at 2, 4 and 8 HPC. The upregulated gene expression of the FCN stomach included complement C3-like (*LOC124627631*) at 2 and 4 HPC, *c7b* and CD59 glycoprotein (*cd59 LOC108280891*) each at 8 HPC. The downregulated gene expression in FCN stomach included defender against cell death 1 (*dad1*) and caspase activity and apoptosis inhibitor 1 (*caap1 LOC108260930*) each at 2 and 8 HPC.

The NCF stomach upregulated genes included *c3 LOC108258708* and *c7b* at 8 HPC, and most downregulated gene cell death inducing DFFA like effector c (*cidec*) a 4 and 8 HPC and complement component 6 (*c6*) at 2 and 4 HPC. The NCN stomach identified upregulated genes that included BCL2 binding component 3 (*bbc3*) and *cideb* each at 2, 4 and 8 HPC. Downregulated genes included *dad1* and NADPH oxidase 1 (*nox1*) each at 2, 4 and 8 HPC.

We also examined the expression of individual DEGs in the intestine that were associated with cell death (**Figure 8B**, **Supplementary Table S4**). The upregulated gene expression of the FCF intestine included BCL2 interacting killer (*bik*) and BCL2 binding component 3 (*bbc3*) each at 2 and 8 HPC and CD59 glycoprotein (LOC108280891) at 8 HPC. Downregulated expression had the *c6* at 4 HPC and *dad1* at 2 and 4 HPC. The upregulated gene expression of the FCN stomach also included BCL2 interacting killer (*bik*) and BCL2 binding component 3 *(bbc3*) each at 2 and 8 HPC and *cd59 LOC108280891* at 8 HPC. The NCF intestine upregulated genes included complement C3-like (LOC124627631) at 2 and 8 HPC, complement C3 (LOC108258708) at 4 and 8 HPC and BCL2 binding component 3 (*bbc3*) at 2, 4 and 8 HPC. Downregulated genes included defender against cell death 1 (*dad1*) at 2, 4 and 8 HPC and superoxide dismutase 1, soluble (*sod1*) at 2 and 4 HPC. NCN intestine upregulation included BCL2 interacting killer (*bik*) and BCL2 binding component 3 (*bbc3*) at 2, 4 and 8 HPC. Downregulation included complement component 1, q subcomponent binding protein (*c1qbp*) defender against cell death 1 (*dad1*) each at 2, 4 and 8 HPC.

#### 3.3.5 Validation of RNA sequencing

Reverse transcription qPCR was conducted on six intestinal RNA samples from the control and 8 HPC samples for each treatment (31). Five genes involved in pathogen recognition, inflammatory and immune response were chosen for validation of the RNA sequencing analysis. All RT-qPCR data correlated with RNA sequencing data (**Supplementary Table S5**).

## 4 Discussion

### 4.1 Impact of skin injury and feeding on survivability of MAS-infected channel catfish

In this study, we demonstrate the significant influence of Af clipping and feeding status on the progression of vAh-induced MAS in channel catfish. Survival trends among the challenged fish led to mortality at 8 HPC and then peaked at 24 HPC, which aligns with previous work (12, 13), and established that this experimental design is suitable for these analyses. Teleost fish skin damage is widely acknowledged as a key entry point for many bacterial pathogens (15, 32, 33). Recent work has shown that Af clipping serves as an effective method for creating standardized wounds, allowing researchers to examine vAh virulence in catfish (12). In this study, fish that were injured and unfed (FCN) demonstrated the lowest survival rate of 23%, emphasizing the severe impact of combining injuries with lack of food on survival. Likewise, the survival rate for fish that were injured and fed (FCF) was slightly higher at 30%. These observations are consistent with previous work that demonstrated fish skin damage, suggesting that disruption of skin integrity increases the vulnerability to MAS infection (34).

Additionally, the fed status of challenged fish, may have influenced their ability to counter infections (35), and this could partially explain why fish in the injured but unfed group (FCN) required longer to initiate an immune response against infection. The use of commercial feeds in aquaculture systems contributes to the bacterial diversity within aquatic environments (36). Moreover, as fish become lethargic during the progression of the disease state, the presence of feed facilitates the proliferation of vAh in water/the environment, as uneaten feed may create an ideal environment for vAh growth and, if using medicated feed, potentially further the development of antibiotic resistant *Aeromonas* strains (37). This finding sheds light on the conditions that promote MAS outbreaks in aquaculture settings and underscores the modulatory role of feed availability in the pathogenesis of MAS infections (38).

### 4.2 Tissue responses in the gastrointestinal tract of MAS-infected channel catfish

The external lesions observed in this study, including bilateral exophthalmia and redness around the gills, mouth, head, and fins (**Figure 2**), align with previously described symptoms of vAh-induced MAS (7, 14, 39). Similar external manifestations, such as hemorrhagic dermatitis and ulcerations, have been reported as defining characteristics of vAh infections in farmed catfish during MAS outbreaks in Mississippi and Alabama (7). Gastrointestinal damage, characterized by epithelial necrosis and lymphocytic infiltration, aligns with findings from earlier studies. Extensive necrosis of the gastrointestinal mucosa and accumulation of necrotic debris in MAS-infected catfish was documented (14), supporting the progression of intestinal damage seen in this study. Notably, the fed groups (FCF, NCF) exhibited more severe lesions than the unfed groups, suggesting that a greater amount of bacteria may have been present in these fish (**Supplementary Figures S1**, **S2**). Internally, significant engorged gastric arteries were observed in the fed groups, consistent with other findings (7). These observations suggest a temporary increase in splanchnic blood flow after feeding, which facilitates the gastrointestinal tract’s enhanced metabolic and absorptive processes (40, 41). In contrast, the unfed groups displayed less pronounced changes in the intestines and stomach, highlighting a potential link between fed status and vascular response during infection. The absence of recent feeding may limit the blood flow transporting vAh cells, mitigating the inflammatory responses that contribute to tissue damage. The presence of vAh cells in the blood between 1 and 24 HPC, with a rapid increase between 1 and 8 HPC (13), combined with heightened splanchnic blood flow after feeding, explains the observed gastrointestinal lesions in the fed groups vs the unfed groups. These findings align with previous reports indicating significantly higher concentrations of vAh cells in the stomach and intestines of infected fish (12, 13).

The observations in this study are consistent with MAS and vAh infections in finfish; however, differences in pathology between fed and unfed groups provide valuable insights into how fed status influences disease severity. These findings show that feeding intensifies gastric and enteric damage, suggesting that nutrient availability modulates the distribution and severity of lesions. These findings are in line with previous studies that identified stressors such as feeding and environmental factors as contributors to the progression of MAS outbreaks (14). Further, the progression in lesion severity observed in this study provides novel insights into the temporal dynamics of MAS in channel catfish. Using a semiquantitative grading system in this study further underscores significant differences at 4 HPC between the fed and unfed treatment groups.

### 4.3 Global gene expression responses in the gastrointestinal tissues of MAS-infected channel catfish

#### 4.3.1 Gene ontology enrichment

Our global gene expression study identified the top GO terms shared among each of the individual treatment groups where the majority of them dealt with fundamental cellular processes which is consistent with what others have shown in catfish when infected with *Aeromonas* spp (42–44). Interestingly, one of the GO terms associated with one or both tissues in each treatment was for oxidoreductase activity (GO:0016491) which is associated with reactive oxygen species (ROS) synthesis (**Tables 2**, **3**, **Supplementary Table S3**). We also identified several select GO terms associated with neutrophil-mediated killing, activation, response and regulation of ROS processes (45–47). These results suggest that most activated immune processes have predominantly occurred in the fin clipped stomach regardless of feeding status. Overall, these findings seem indicative of normal cellular processes and have not demonstrated differences in a disease state between the groups as observed with the survival analysis.

#### 4.3.2 Inflammatory response

Cytokines have essential roles in the innate immune system and inflammation response that is largely conserved across taxa (48). In this study, we observed that interleukins were broadly modulated during infection, with *il-1β* showing robust upregulation in both stomach and intestinal tissues, particularly in the fin clipped groups. In the FCF stomach, *il-1β* expression was elevated across all time points (2, 4, and 8 HPC), suggesting early and sustained pro-inflammatory signaling (47–49). Similarly, in the intestine, two distinct *il-1β* paralogs (*LOC100304696* and *LOC100304697*) were markedly upregulated at 8 HPC in FCF and NCF groups. Collectively, these data support an active interleukin-mediated response, particularly in the stomach, where inflammatory signaling is likely more immediate and intense. The intestine exhibited more temporally restricted interleukin responses, with fewer consistent upregulations. Similarly, in the intestine, two distinct *il-1β* paralogs (*LOC100304696* and *LOC100304697*) were markedly upregulated at 8 HPC in FCF and NCF groups. Collectively, these data support an active interleukin-mediated response, particularly in the stomach, where inflammatory signaling is likely more immediate and intense. The intestine exhibited more temporally restricted interleukin responses, with fewer consistent upregulations (**Supplementary Table S4**).

In parallel, TNF superfamily members displayed notable expression patterns, particularly through differential regulation of TNF receptor superfamily members (e.g., *tnfrsf11a*, *tnfrsf14*, *tnfrsf6b*). In both FCF and FCN stomach tissues, *tnfrsf11a* and *tnfrsf14* were persistently downregulated, a pattern also observed for *tnfrsf14* in the NCF and NCN groups, though with lower magnitude. These trends suggest possible dysregulation or negative feedback within the TNF axis during peak inflammation. Interestingly, the intestine displayed TNF ligand and receptor modulation, with modest upregulation and limited downregulation for *tnfrsf14*. These findings point to a stomach-centric role of TNF signaling dysregulation, potentially contributing to epithelial apoptosis and tissue damage (50, 51).

Chemokine signaling further revealed divergent tissue responses, with the stomach exhibiting widespread upregulation of chemokines such as *cxcl18b*, *cxcl14*, *cxcr4b*, and others across all post-challenge time points, consistent with enhanced leukocyte recruitment and local inflammation (52–54). Notably, *cxcl18b* and *cxcl14* were also upregulated in the intestine, albeit at 4 and 8 HPC in the fed groups, suggesting slightly delayed chemotactic signaling. In contrast, chemokines such as *cxcl12a* and *cxcl12b* were consistently downregulated in the intestine across multiple groups and time intervals, indicating a disruption in homeostatic or anti-inflammatory chemokine gradients. The broader and earlier chemokine activation in the stomach suggests that this organ could be the primary site of immune cell infiltration and inflammatory signaling during MAS infections in channel catfish.

Chemokine signaling further revealed divergent tissue responses, with the stomach exhibiting widespread upregulation of chemokines such as *cxcl18b*, *cxcl14*, *cxcr4b*, and others across all post-challenge time points, consistent with enhanced leukocyte recruitment and local inflammation (52–54). Notably, *cxcl18b* and *cxcl14* were also upregulated in the intestine, albeit at 4 and 8 HPC in the fed groups, suggesting slightly delayed chemotactic signaling. In contrast, chemokines such as *cxcl12a* and *cxcl12b* were consistently downregulated in the intestine across multiple groups and time intervals, indicating a disruption in homeostatic or anti-inflammatory chemokine gradients. The broader and earlier chemokine activation in the stomach suggests that this organ could be the primary site of immune cell infiltration and inflammatory signaling during MAS infections in channel catfish.

#### 4.3.3 Complement and apoptotic responses

Complement systems alert the host to the presence of potential pathogens, serving as a crucial component of the innate immune response, facilitating pathogen recognition, opsonization, and lysis while bridging innate and adaptive immunity (55). The status of cell homeostasis revealed in our study showed that complement activation was prominently upregulated in the stomach, especially in the fin-clipped groups (FCF and FCN). Notably, components such as *complement C3* and *C7b*, as well as *C5a receptor 1*, were elevated in the FCF and FCN stomach by 8 HPC, suggesting strong opsonophagocytic and inflammatory signaling (56, 57). Similarly, the FCF and FCN stomach at 8 HPC showed upregulation of *C3-like* variants and terminal complement regulators such as *CD59*, further indicating complement system engagement. These patterns suggest that the complement cascade is rapidly mobilized in the stomach in response to vAh, potentially contributing to tissue inflammation. In contrast, the intestines showed upregulation of *c3* by 8 HPC within the unfed groups (FCN and NCN), suggesting that increased complement cascade activity within the intestines of unfed channel catfish in response to vAh.

Moreover, the expression of genes regulating apoptotic signaling showed widespread patterns, particularly in the stomach and intestine of all challenged groups. In the stomach, pro-apoptotic regulators such as *bbc3*, *bik*, and *cideb* were upregulated across time points, concurrent with downregulation of anti-apoptotic genes like *arel1*, *dad1*, and *caap1 LOC108260930* (58, 59). The overexpression of pro-apoptotic genes, such as *bbc3*, *bik*, and *cideb*, prevented epithelial apoptosis, which is consistent with the histological findings of the absence of apoptotic tissues within the stomach. Additionally, this overexpression decreases epithelial apoptosis through tight junction alterations which could be responsible for better survival rates in the unfed groups within the first 8 HPC (60). Interestingly, the intestine exhibited a broader apoptotic signature across all treatment groups. Pro-apoptotic genes such as *bbc3*, *bik*, and *cidec* were upregulated in the FCF, FCN, NCF, and NCN intestines, while anti-apoptotic genes, including *dad1*, *sod1*, and *c1qbp* were persistently downregulated. These observations suggest a progressive apoptotic process that occurs in the intestine, regardless of the host’s nutritional or physical condition. Again, these combined results indicate that the fin-clipped stomach is actively working to facilitate an innate immune response to the pathogen, while the intestine is much less active (**Supplementary Table S4**). The overall substantial increase in apoptotic activity in the intestine, regardless of clipped or fed status, would suggest that these cells are dead or dying because of a limited innate response.

## 5 Conclusion

In conclusion, this study demonstrates that both skin injury and fed status are critical modulators of host susceptibility, immunopathology, and gastrointestinal immune responses during *A. hydrophila*-induced MAS in channel catfish. Fin clipping exacerbated mortality and inflammation, especially when combined with feeding, highlighting the compounding effects of physical damage and metabolic activity on disease outcome. Gastrointestinal lesions and transcriptomic data collectively indicate that the stomach serves as a principal site of immune activation, with enhanced complement activation, cytokine signaling, and chemokine recruitment contributing to the observed pathology. In contrast, the intestine exhibited a more restrained inflammatory response but displayed pervasive apoptotic gene expression as the infection progressed over time, suggesting widespread epithelial damage and compromised cellular homeostasis. These tissue-specific immune signatures provide insight into the spatial dynamics of host-pathogen interactions in vAh infections. Furthermore, the data underscore the importance of early inflammatory regulation, as excessive or dysregulated responses correlated with increased mortality. Overall, these findings advance our understanding of how host factors such as injury and fed status modulate innate immunity and disease severity in aquaculture settings. This knowledge is essential for developing evidence-based strategies to mitigate MAS outbreaks, emphasizing the importance of minimizing physical stress and managing feeding regimens during high-risk periods. Withholding feed is a management strategy for enteric septicemia of catfish, but has adverse effects on innate immunity and mortality against columnaris disease (61–64). This current work would suggest that even a brief fasting period (overnight) prior to *v*Ah has limited impact on organosomatic indexes and may influence disease susceptibility, as reflected by reduced intestinal pathology and earlier immune activation. However, the optimal duration and physiological thresholds of pre-challenge fasting remain undefined. Future studies should define optimal fasting durations that balance disease resistance with growth performance, incorporate microbiome profiling to uncover gut microbial shifts following nutritional modulation, and evaluate the long-term immunological and physiological impacts of repeated or prolonged feed withholding in aquaculture systems.

## Data Availability

The RNA sequencing datasets generated for this study can be found in the NCBI Gene Expression Omnibus (GEO) repository and can be accessed under accession numbers GSE281206 (intestine) and GSE281208 (stomach). All other data that support the findings of this study have been included in the manuscript and Supplementary Materials.
