# Supplementary material for: Feed status and skin injury modulate immunopathology, global gene expression, and survival in channel catfish during virulent Aeromonas hydrophila infection
Source: Front Immunol. 2025 Jul 22;16:1642531. doi: 10.3389/fimmu.2025.1642531 (PMC12321539; doi:10.3389/fimmu.2025.1642531)
Supplement: Supplementary file 6 [file DataSheet1.docx]

Supplementary Material

**Feed Status and Skin Injury Modulates Immunopathology, Global Gene Expression Changes, and Survival in Channel Catfish During Virulent *Aeromonas hydrophila* Infection**

**Yesutor K. Soku^1†^, Miles D. Lange^2*†^, Jason W. Abernathy^2^, Nithin M. Sankappa^2,3^, Craig Shoemaker^2^, Karl Hayden^1^, Linnea Andersen^2^, Ida Phillips^1,4^, Toufic Nashar^1^, Temesgen Samuel^1^, Abdelrahman Mohamed^1*^**

^1^ Department of Pathobiology, College of Veterinary Medicine, Tuskegee University, Tuskegee, Alabama, United States of America

^2^ United States Department of Agriculture, Agricultural Research Service, Aquatic Animal Health Research Unit, Auburn, Alabama, United States of America

^3^ Oak Ridge Institute for Science and Education (ORISE), ARS Research Participation Program, Oak Ridge, Tennessee, United States of America

^4^ Rollins Laboratory, Raleigh, North Carolina, United States of America

^5^ Department of Biological Sciences, Auburn University, Auburn, AL, United States of America

# Supplementary Figures

D)

C)

B)

A)


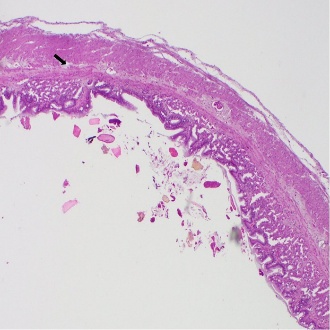

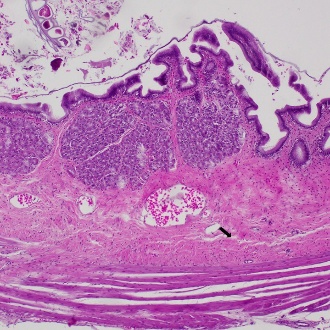

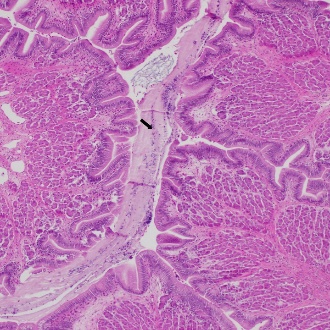

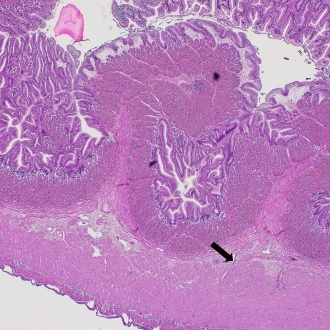


H)

G)

F)

E)


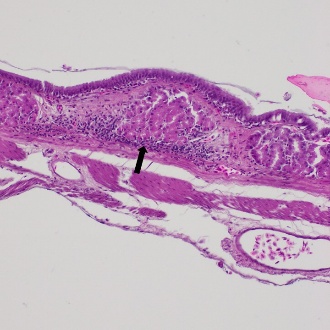

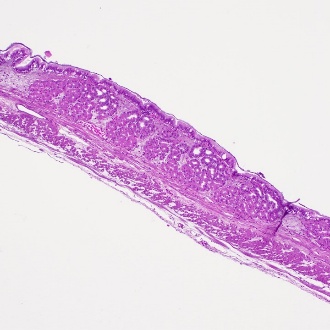

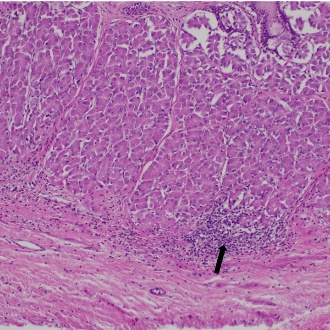

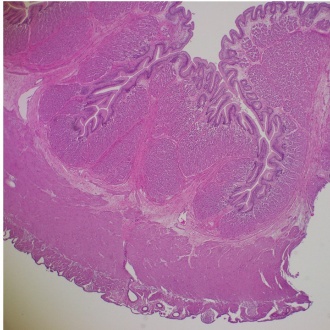


L)

K)

J)

I)


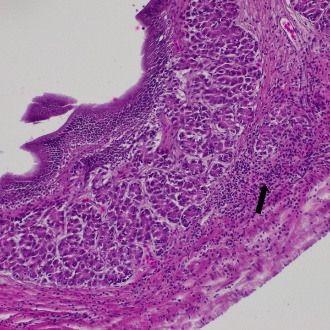

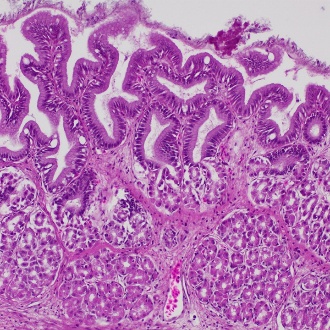

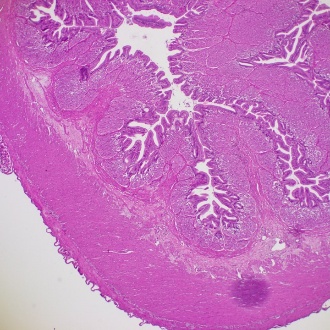

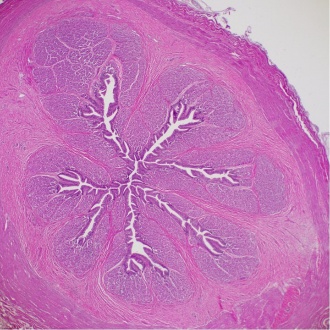


**Supplementary Figure 1:** Photomicrographs of the stomach of infected *Ictalurus punctatus.* At 2 HPC (A) Minimal edema (arrow) was in the lamina propria of the stomach in the FCF group,100x, H&E, FCF. (B) Minimal to mild edema (arrow) was detected in the lamina propria of the stomach in the NCF group, 100x, H&E. (C) Mild to moderate edema in the lamina propria and submucosa of the stomach with cellular debris within the lumen in the FCN group, 200x, H&E. (D) Mild edema (arrow) in the lamina propria and submucosa of the stomach in the NCN group, 100x, H&E. At 4 HPC (E) Moderate lymphoplasmacytic and granulocytic infiltration (arrow) with mild and moderate edema in the FCF group, 100x, H&E. (F) Moderate edema within the lamina propria with a few lymphocytes and plasma cells in the NCF group, 100x, H&E. (G) Mild lamina propria and submucosal edema with lymphoplasmacytic aggregation (arrow) in the FCN group, 400x, H&E. (H) Mild lamina propria and submucosal edema in the NCN group, 100x, H&E. At 8 HPC (I) Edema within the lamina propria with increased lymphocytes and plasma cells (arrow) in the FCF group, 200x, H&E. (J) Multifocal erosions (arrow) to superficial epithelium with diffuse moderate to severe transmural edema in the NCF group, 400x, H&E. (K) Mild to moderate lamina propria and submucosal edema in the FCN group, 100x, H&E. (L) Minimal to mild lamina propria and submucosal edema in the NCN group, 100x, H&E.

D)

C)

B)

A)


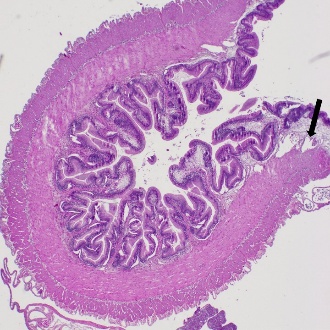

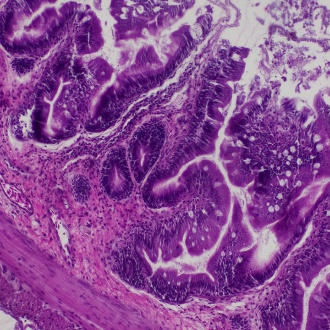

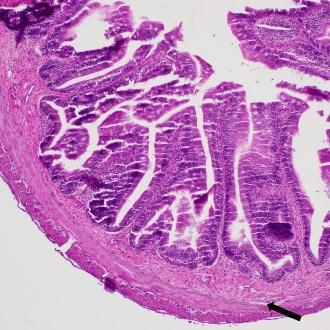

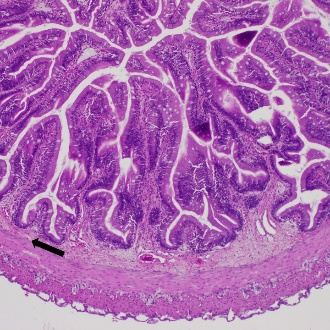


H)

G)

F)

E)


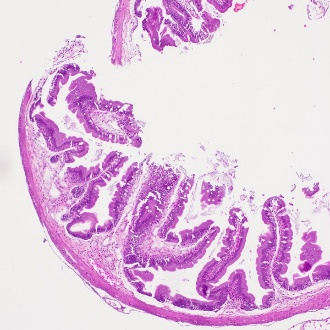

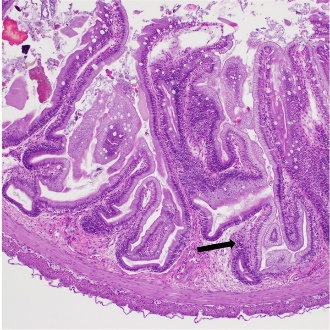

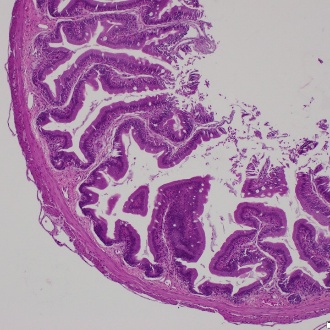

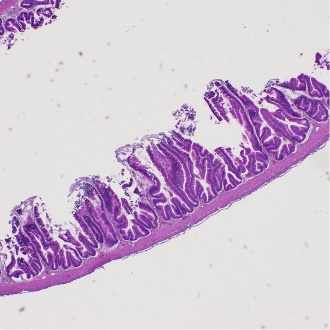


L)

K)

J)

I)


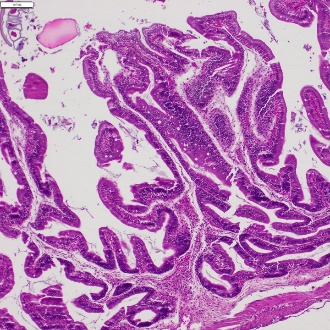

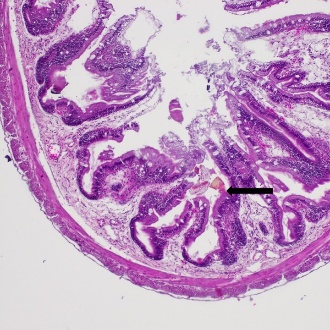

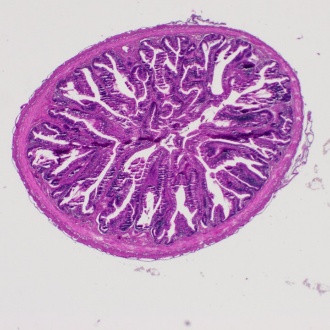

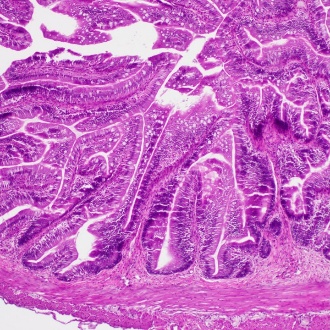


**Supplementary Figure 2:** Photomicrographs of the intestines of infected *Ictalurus punctatus*. At 2 HPC (A) Mild to moderate expansion of the lamina propria by edema and dilation of lymphatic vessels, minimal lymphocytes and plasma cells have also been infiltrated (arrow), 100x, H&E, FCF. (B) Minimal to mild edema within lamina propria, 400x, H&E, NCF. (C) Minimal (No significant) findings, 200x, H&E, FCN. (D) Mild lamina propria and submucosal edema (arrow), 200x, H&E, NCN. At 4 HPC (E) More severe edema within the lamina propria with a few lymphocytes and plasma cells, 100x, H&E, FCF. (F) Moderate edema within the lamina propria with a few lymphocytes and plasma cells (arrow), 100x, H&E, NCF. (G) Minimal (No significant) findings, 200x, H&E, FCN. (H) Mild lamina propria and submucosal edema, 100x, H&E, NCN. At 8 HPC (I) Moderate edema within the lamina propria with a few lymphocytes and plasma cells (arrow), 200x, H&E, FCF. (J) Degenerative changes to the superficial epithelium (arrowhead) with diffuse severe transmural edema and hemorrhage (arrow), 200x, H&E, NCF. (K) Minimal (no significant) findings, 100x, H&E, FCN. (L) Minimal to mild lamina propria and submucosal edema, 200x, H&E, NCN.
